# General anesthesia of a Japanese infant with Barber-Say syndrome: a case report

**DOI:** 10.1186/s40981-016-0033-x

**Published:** 2016-06-02

**Authors:** Eisuke Hamaguchi, Yasuo M. Tsutsumi, Katsuyoshi Kume, Yoko Sakai, Nami Kakuta, Yuta Uemura, Shinji Kawahito, Katsuya Tanaka

**Affiliations:** Department of Anesthesiology, Tokushima University Hospital, 2-50-1 Kuramoto, Tokushima, 770-8503 Japan

**Keywords:** Barber-Say syndrome, Macrostomia, Cutis laxa, General anesthesia

## Abstract

**Background:**

Barber-Say syndrome (BSS) is a very rare congenital disorder characterized by macrostomia, cutis laxa, and other features. We report our experience of performing general anesthesia on a Japanese child with BSS.

**Case presentation:**

A bilateral repair of the corners of the mouth under general anesthesia was planned for an 18-month-old male with macrostomia; the child was 75 cm in height and weighed 9.9 kg. As insertion of the peripheral intravenous catheter was difficult, it was inserted before the surgery by a pediatrician. The patient wore a mask and was ventilated manually after loss of consciousness with intravenous anesthesia. A mask for adults provided a superior fit and was effective in preventing air leakage from the corners of the mouth. After rocuronium was administered, the larynx was spread with a Macintosh laryngoscope. There was no laryngeal anatomical abnormality, and tracheal intubation was readily possible. The operation was completed without incident. Stiffening of both arms occurred for several seconds one hour after the operation ended, but the patient did not develop other complications.

**Conclusions:**

Mask ventilation and the insertion of an intravenous catheter may be difficult in the general anesthesia of patients with BSS, and anesthetic management requires caution.

## Background

Barber-Say syndrome (BSS) is characterized by unique congenital malformations, including macrostomia, redundant skin, and other features [1]. It is a very rare syndrome, and only several dozen cases have been reported thus far, no previous report on the general anesthesia of a patient with BSS has been published to our knowledge. Following our experience of performing plastic surgery under general anesthesia for a child with BSS, we report the details.

## Case presentation

**Fig. 1:**
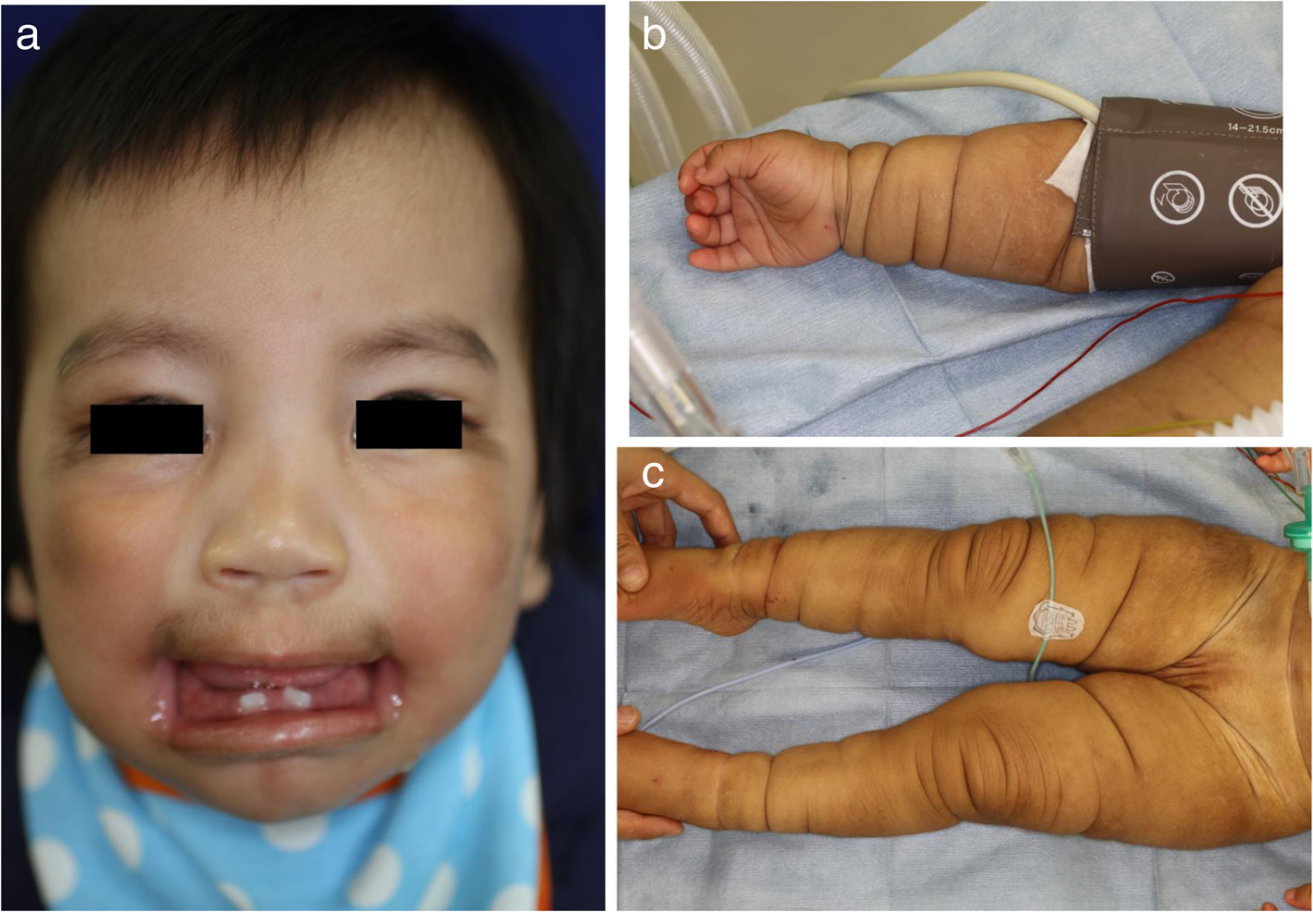
Clinical photography of the patient before surgery. **a**) Characteristic forms of BSS are shown, including macrostomia, gingival dysplasia, and ocular telecanthus. **b**-**c**) The skin folds on the limbs were remarkable

A Japanese male was born with a length of 50.4 cm and a weight of 3390 g by vacuum extraction, and his Apgar scores were 9 and 9 at 1 and 5 min after birth, respectively. Characteristic forms of abnormality were observed such as macrostomia, gingival dysplasia, ocular telecanthus, hypertrichosis, dark brown and dry skin with redundant folds on the limbs, and hypospadias. From these malformations, he was clinically diagnosed with BSS by pediatricians based on searching both the Pub-Med database (http://www.ncbi.nlm.nih.gov/pubmed), and the University of Ryukyu Database for Malformation Syndromes (http://becomerich.lab.u-ryukyu.ac.jp/) [2]. Plastic surgery (repair of the corners of the mouth, shortening of the lingual frenulum, and shortening of the upper lip frenulum) for macrostomia was planned. The patient was 1 year and 6 months old, his height was 75 cm, and his weight was 9.9 kg just before the operation. On physical examination, the previously observed findings of macrostomia and redundant skin on the limbs were recognized (Fig. 1), but another physical abnormality was not observed, including neurologic abnormality. Furthermore, he had no episode of a seizure before the surgery. A 12-lead electrocardiogram, chest radiogragh, and blood tests were carried out as preoperative examinations, and no abnormality was indicated.

### Management of general anesthesia

**Fig. 2:**
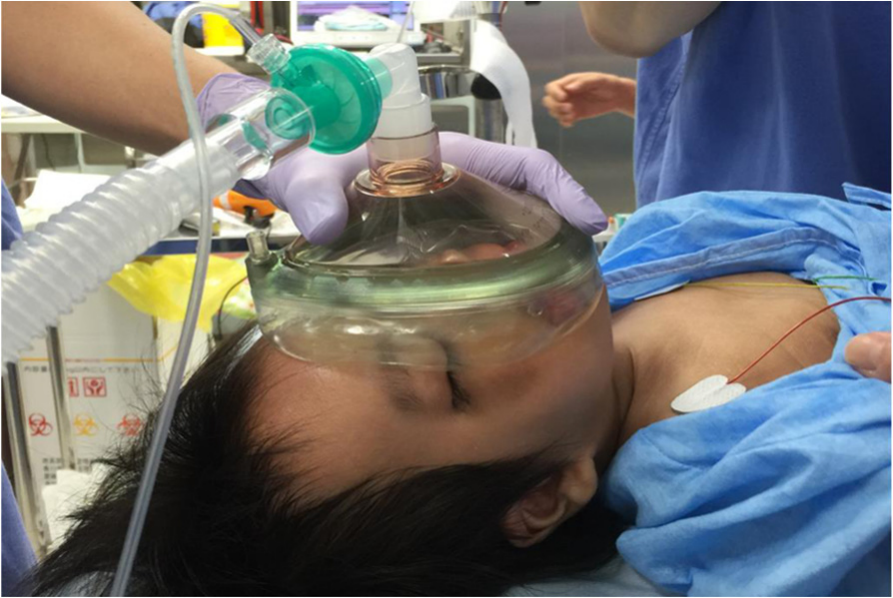
Photography of manual ventilation of the patient. A mask for large adults (The Original King Mask™, size 6) was suitable to cover both corners of the mouth

Difficulty inserting of the intravenous catheter was expected because of the skin folds on the limbs; therefore, a 24-G line had been inserted in the right forearm by a pediatrician on the day before the operation. For the prevention of difficulty to ventilate during the induction of anesthesia, we stood by with a difficult airway management cart and a bronchial fiberscope. In addition, we prepared masks of various sizes for manual ventilation. Premedication was not performed before general anesthesia. On arrival in the operating room, pulse oximetry, electrocardiography, and non-invasive blood pressure monitoring were established. Manual mask ventilation with 100 % oxygen was performed after the patient lost consciousness after the administration of 3 mg of midazolam intravenously. Because air leakage was observed from both corners of the mouth with a mask for toddlers (The Original King Mask™, size 3; King Systems Corporation, Noblesville, IN, USA), it was switched to a mask for large adults (The Original King Mask™, size 6; King Systems Corporation), which provided a good fit and made manual ventilation easy (Fig. 2). Tracheal intubation was performed with an oral RAE Tracheal Tube Cuffless^TM^ with an inner diameter of 4.5 mm (Covidien, Dublin, Ireland) by Macintosh laryngoscope three minutes after the inhalation of 8 % sevoflurane and the administration of 10 mg of rocuronium intravenously. The Cormack and Lehane classification was grade 1, and there were not any laryngeal anatomical abnormality or stenosis. General anesthesia was maintained with 2 % sevoflurane inhalation and 0.2–0.25 μg/kg/min of remifentanil intravenously, and vital signs were kept stable. Several anesthesiologists tried to place an additional intravenous catheter, but gave up because of difficulties. The operation was finished without any incident, and the patient was extubated in the operating room after the administration of acetaminophen 75 mg and sugammadex 80 mg intravenously. A wakening was prompt, signs of upper airway occlusion were not observed, and the volume of air entering the lungs was good. The operation duration was 235 min, the anesthesia duration was 334 min, there was a small amount of bleeding, and the urinary output was 80 mL. One hour after surgery, two attacks of stiffening of the arms and rolling of the eyes for several seconds were noted. The attacks resolved naturally and did not recur. Epilepsy waves were detected by electroencephalography five days after the surgery, and he has been followed up carefully. In addition, no other complication occurred, and the patient was discharged from our hospital eight days after surgery.

## Discussion

BSS was reported first in 1986 by Say, Barber, et al. [3]. Only several dozen people have been reported as having BSS previously, and our patient is the first case of BSS in Japan [2]. Only one case suggested that BSS was inherited as an autosomal-recessive trait because the parents were consanguineous [4]; however, a recent study revealed that autosomal-dominant TWIST2 mutations cause BSS by inducing protean effects on the transcription factor’s DNA binding [5]. In our patient, a G-banded chromosome analysis revealed a 46, XY karyotype. In the past, physical and mental retardation [3, 6, 7] and transient hypogammaglobulinemia [3] were reported. Otherwise, complications of congenital cardiac anomaly, lung disturbance, or laryngeal stenosis leading to difficult intubation have not been demonstrated in BSS.

It is necessary to prepare beforehand for difficulty in maintaining an airway during the induction of general anesthesia in patients with BSS. Generally, the use of a supraglottic device (SGD) and bronchial fiberscope have been shown to be effective for airway maintenance in pediatric anesthesia when there is concern for difficult airway [8]. Jain et al. [9] reported that covering both corners of the mouth with surgical paper tape was effective in preventing air leakage from the mouth in pediatric anesthesia with macrostomia. On the other hand, there is a report that it was effective to use an airway catheter together with a SGD at the induction of anesthesia in patients with macrostomia [10]. During anesthesia in our case, air leakage from both corners of the mouth that occurred with a mask for toddlers disappeared with a mask for adults was used, suggesting that it is important to confirm that the size of the mask is large enough to cover the corners of the mouth during the preoperative physical examination.

Difficulty inserting an intravenous catheter because of redundant skin and hypertrichosis on the limbs would be a common problem in general anesthesia for patients with BSS. In our case, senior anesthesiologists tried to insert an additional intravenous catheter, but the attempts ended in failure. It seems to be essential that a pediatric anesthetic expert insert an intravenous catheter before the induction of general anesthesia to avoid slow induction in patients with BSS.

After surgery, attacks of stiffening of both arms occurred for a short time. Epilepsy waves were later detected by electroencephalography, and these attacks might have been due to epilepsy. However, epileptogenic effects have often been reported in general anesthesia with sevoflurane [11], and would not be able to be distinguished from a characteristic epilepsy attack. It is difficult to judge at present which volatile and intravenous anesthetics are most appropriate for the general anesthesia of patients with BSS, but it might be reasonable to avoid a volatile anesthetic in children with BSS who have experienced convulsions in the past.

This is the first report of general anesthesia of a patient with BSS. It would be expected that accumulating cases about the general anesthesia of patients with BSS, including the choice of appropriate general anesthetics and better airway management, be performed in the future to ensure the perioperative safety of patients with BSS.

## Conclusions

We performed general anesthesia on a child with BSS. In the general anesthesia of patients with BSS, it is necessary to note air leakage during mask ventilation and difficulty in the insertion of a peripheral intravenous catheter.

## Consent

Written informed consent was obtained from the patient’s parents for publication of this case report and any accompanying images. A copy of the written consent is available for review by the Editor-in-Chief of this journal.

## Abbreviations

BSS: Barber-Say Syndrome
SGD: supraglottic device
